# Long non‐coding RNA GAS5 is induced by interferons and plays an antitumor role in esophageal squamous cell carcinoma

**DOI:** 10.1002/cam4.1524

**Published:** 2018-05-09

**Authors:** Jianbing Huang, Yuan Li, Zhiliang Lu, Yun Che, Shouguo Sun, Shuangshuang Mao, Yuanyuan Lei, Ruochuan Zang, Ning Li, Nan Sun, Jie He

**Affiliations:** ^1^ Department of Thoracic Surgery National Cancer Center/Cancer Hospital Chinese Academy of Medical Sciences and Peking Union Medical College Beijing China

**Keywords:** esophageal squamous cell carcinoma, GAS5, interferons, JAK‐STAT pathway, long non‐coding RNA

## Abstract

The long non‐coding RNA GAS5 has been reported as a tumor suppressor in many cancers. However, its functions and mechanisms remain largely unknown in esophageal squamous cell carcinoma (ESCC). In this study, we found that GAS5 was over‐expressed in ESCC tissue compared with that in normal esophageal tissue in a public database. Functional studies showed that GAS5 could inhibit ESCC cell proliferation, migration and invasion in vitro. Further analysis revealed that GAS5 was regulated by interferon (IFN) responses via the JAK‐STAT pathway. Moreover, as an IFN‐stimulated gene (ISG), GAS5 was a positive regulator of IFN responses. The feedback loop between GAS5 and the IFN signaling pathway plays an important antitumor role in ESCC, thus providing novel potential therapeutic targets.

## 1 INTRODUCTION

Esophageal cancer is one of the most common cancers worldwide, ranking eighth in incidence and sixth in mortality among all cancers; most cases of esophageal cancer are esophageal squamous cell carcinoma (ESCC).^1^, ^2^ Esophageal cancer has a poor survival rate due to its aggressive nature and the lack of early diagnostic markers and effective therapies for managing advanced patients.^3^ Thus, a better understanding of the mechanisms underlying the initiation and progression of esophageal cancer is critically important.

Long non‐coding RNAs (lncRNAs) form a vital class of molecules that are involved in numerous biological processes in development and disease^4^, ^5^ and that play an important role in cancer initiation and progression.^6^, ^7^ Many lncRNAs are dysregulated in esophageal cancer,^8^ and some have been developed as potential biomarkers for diagnosis, prognosis and prediction, such as the extensively studied lncRNA HOTAIR (HOX transcript antisense RNA).^9^, ^10^

The lncRNA GAS5 (growth arrest‐specific transcript 5) was identified as a pivotal tumor suppressor in several human cancers, including breast cancer, prostate cancer, lung cancer, and colorectal cancer.^11^ GAS5 can inhibit the proliferation and promote the apoptosis of cancer cells, mainly through repressing steroid receptor‐induced transcriptional activation, acting as an miRNA sponge and directly interacting with functional proteins.^12^ However, the function and mechanisms of GAS5 in ESCC remain largely unstudied.

Here, we found that GAS5 is regulated by the interferon (IFN) response and exerts antitumor effects in ESCC. As an IFN‐stimulated gene (ISG), GAS5 has positive feedback on the IFN signaling pathway. In conclusion, we illustrate the crosstalk between the lncRNA GAS5 and the IFN signaling pathway, thus offering novel potential therapeutic targets for ESCC.

## 2 MATERIALS AND METHODS

### 2.1 Cell culture

The esophageal cancer KYSE30 and KYSE180 cell lines were cultured in RPMI 1640 medium (Catalog No.SH30809.01B, HyClone) at 37°C in a humidified atmosphere at 5% CO_2_. HEK 293T cells were cultured in Dulbecco's modified Eagle's medium (DMEM, Catalog No.SH30022.01B, HyClone). RPMI 1640 medium and DMEM were supplemented with 10% FBS (Catalog No.16000‐044, Gibco) and antibiotics (100 U/mL penicillin and 100 mg/mL streptomycin) (Catalog No.10378016, Thermo Fisher Scientific). All cell lines used in our study were regularly authenticated by short tandem repeat (STR) profiling.

Cells in the control and test groups were harvested at similar confluence. Interferon‐beta (IFN‐β, Catalog No.P01574, R&D) and interferon‐gamma (IFN‐γ, Catalog No.CAA31639, R&D) were used in this study at 1000 IU/mL. In experiments involving ruxolitinib (Catalog No.S1378, Selleckchem), esophageal cancer cells were treated with ruxolitinib (2 μmol/L) for 1 hour prior to the addition of IFN‐β and were then cultured for 72 hours.

### 2.2 RNA extraction and quantitative real‐time PCR (RT‐qPCR)

Total RNA was extracted from cultured cells with the standard TRIzol protocol (Catalog No.15596018, Thermo Fisher Scientific). Cytoplasmic and nuclear RNA were extracted and purified using the Protein and RNA Isolation System (Catalog No.AM1921, Thermo Fisher Scientific) according to the manufacturer's instructions. Complementary DNA (cDNA) was synthesized using a RevertAid First‐Strand cDNA Synthesis kit (Catalog No.K1622, Thermo Fisher Scientific), and quantitative RT‐PCR (RT‐qPCR) was performed in triplicate on an ABI 7900HT Real‐Time PCR thermocycler (Life Technologies). We used the 2^−ΔΔ*Ct*^ method to quantify the relative mRNA expression level, and GAPDH was used as an internal reference. The 2^−ΔΔ*Ct*^ values are log2 transformed when performing statistical tests. At least 2 independent experiments were conducted with a minimum of 2 technical replicates per experiment. All primers used in this study are listed in Table S1.

### 2.3 Knockdown and over‐expression constructs

To obtain stable GAS5 knockdown and over‐expression cell lines, 2 short hairpin RNAs (shRNAs) (shown in Table S1) were cloned into the pLKO.1‐puro vector (Generay, Shanghai, China), and full‐length GAS5 cDNA was inserted into the pCDH‐CMV‐MCS‐EF1‐GFP+Puro (CD513B‐1) vector (Generay, Shanghai, China). Then, the recombinant plasmids and packaging plasmids (pLP1, pLP2 and pLP/VSVG; Thermo Fisher Scientific) were co‐transfected into HEK‐293T cells to obtain infectious lentivirus particles according to the Lipofectamine 3000 instructions (Catalog No.L3000015, Thermo Fisher Scientific). The lentivirus‐containing supernatant was collected at 48 hours and purified with Amicon Ultra‐4 Centrifugal Filter Devices (Millipore). The purified lentivirus supplemented with 5 μg/mL polybrene (Catalog No. 107689, Sigma‐Aldrich) was then used to infect esophageal cancer cell lines. We used corresponding empty vectors as controls. The stable cell lines were obtained after 14 days of selection with puromycin. The knockdown and over‐expression efficiencies were determined by detecting GAS5 mRNA levels via RT‐qPCR.

### 2.4 Cell proliferation, migration and invasion assays

To compare the proliferative abilities of stably transfected cells, CCK8 assays (Dojindo, Kumamoto, Japan) were performed according to the manufacturer's instructions. Briefly, cells (1500 cells/well for KYSE30 and 3000 cells/well for KYSE180) were seeded into 96‐well plates, and OD values were measured with a SpectraMax190 (Molecular Device, USA) at 0, 24, 48, 72, 96, and 120 hours after cell seeding. The fold‐change in OD value relative to 0 hour was obtained as an indicator of cell number. To evaluate the migration and invasion abilities of stably transfected cells, transwell assays (Corning, 8.0‐μm pores) were performed with or without matrigel coated on the upper chamber. Briefly, cells (5 × 10^4^ for migration and 2 × 10^5^ for invasion) were seeded into the upper chamber in 200 μL of serum‐free RPMI 1640 medium, and 750 μL of RPMI medium containing 20% FBS was placed in the lower chamber. We fixed and stained the cells that had translocated to the lower side of the membrane after 24 hours. Five random areas were imaged, and cells were counted at 100‐fold magnification.

### 2.5 Chromatin immunoprecipitation (ChIP)

The esophageal cancer KYSE30 cell line was treated with recombinant IFN‐β (Catalog No.P01574, R&D) or phosphate buffer saline (PBS) for 6 hours before ChIP assays were performed with the SimpleChIP^®^ Enzymatic Chromatin IP Kit (#9003, CST) according to the manufacturer's instructions. DNA was purified from the chromatin fraction precipitated with an anti‐SATA1 antibody (Catalog No.14994T, CST) or control (IgG) and was detected with RT‐qPCR using promoter‐specific primers. The enrichment intensity was normalized to input in anti‐STAT1 and IgG group first. Then, the fold‐change of enrichment intensity with anti‐STAT1/IgG was shown in IFN‐β‐treated and PBS‐treated group, respectively.

### 2.6 Public database

The GSE53624 data set was processed as previously described.^13^ The normalized median‐centered expression intensity in the Oncomine (http://www.oncomine.org) microarray GSE23400 data set was downloaded from GEO https://www.ncbi.nlm.nih.gov/geo/). The GAS5 probe with the largest mean intensity was retained.

The transcription factor binding prediction databases PROMO (http://alggen.lsi.upc.es/cgi-bin/promo_v3/promo/promoinit.cgi?dirDB=TF_8.3) and Jaspar (http://jaspar.genereg.net/) were used with default parameters. The sequence ranging from 2000‐bp upstream to 100‐bp downstream of the GAS5 transcription start site was considered the promoter region.

### 2.7 Statistical analysis

Statistical analysis was performed using GraphPad Prism 7.0. All data were presented as the mean ± standard deviation. Data involving ratio results were all log2 transformed when performing statistical tests. Student's *t*‐test (2‐tailed) was used to analyze experiments involving only 2 groups and one‐way ANOVA was used to analyze experiments involving 3 or more groups. Overall survival was estimated using the Kaplan‐Meier method and compared with the log‐rank test. Differences were considered significant at *P* < .05.

## 3 RESULTS

### 3.1 Expression and prognosis analysis of the lncRNA GAS5 in ESCC

**Figure 1:**
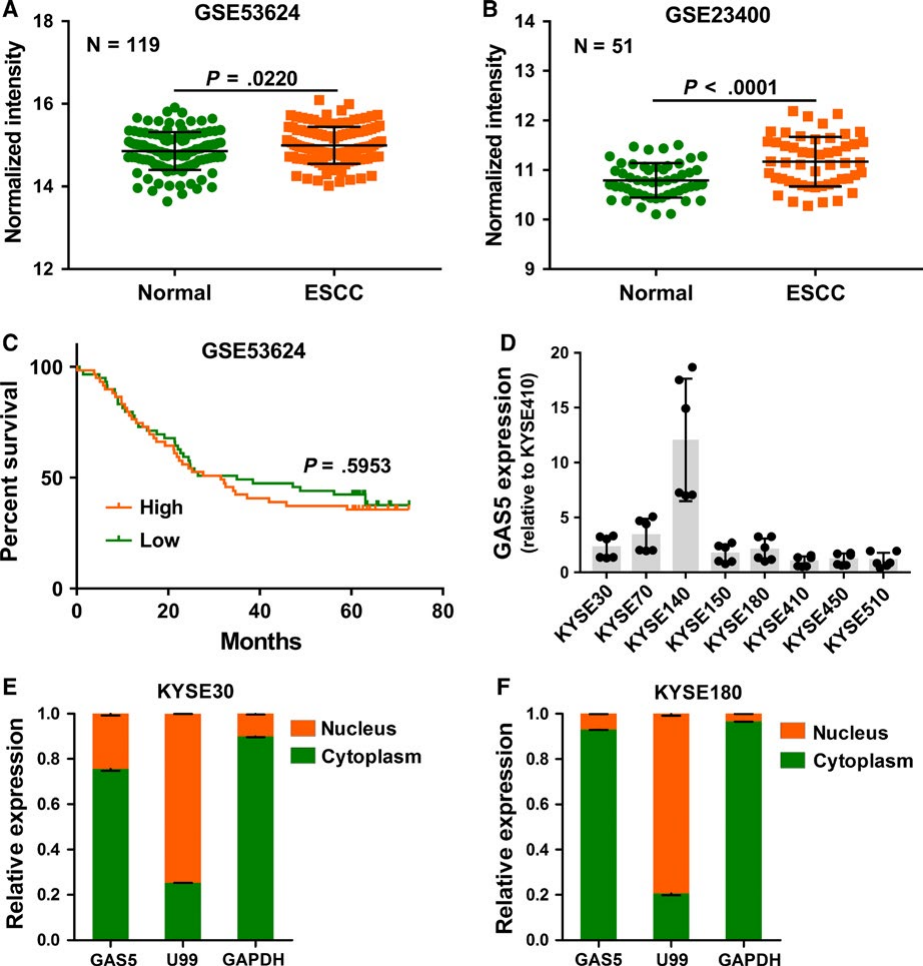
Expression and prognosis analysis of the lncRNA GAS5 in ESCC. A,B, Expression level of GAS5 in the GSE53624 and GSE23400 data sets. “Normal” denotes normal adjacent tissue. Data are presented as the mean ± SD. *P* values are obtained based on Student's *t*‐test (2‐tailed). C, Kaplan‐Meier curve of overall‐survival in ESCC patients with high lncRNA GAS5 level (n = 59) and low lncRNA GAS5 level (n = 59) (*P* = .5953) in data set GSE53624. Patients are split based on median. *P* value is obtained based on Log‐rank test. D, Expression levels of GAS5 in 8 ESCC cell lines detected by RT‐qPCR. E,F, Subcellular localization of GAS5 in KYSE30 and KYSE180 cells. The nuclear marker U99 and the cytoplasmic marker GAPDH were chosen as controls. RT‐qPCR results represent the average of at least 2 independent experiments with a minimum of 2 technical replicates per experiment. The results are presented as the mean ± SD

We first analyzed the GAS5 expression level in our previous ESCC microarray data (GSE53624)^13^ and an Oncomine ESCC data set (GSE23400). The results showed that GAS5 was over‐expressed in ESCC compared with normal esophageal tissue (Figure 1A,B). We next collected the clinical data in data set GSE53624. To analyze the prognostic value of GAS5, we split the patients based on median expression of GAS5. Patients with high GAS5 expression and low GAS5 expression showed no significant difference in overall survival (Figure 1C). We then detected GAS5 expression in 8 different ESCC cell lines (Figure 1D). The location of a lncRNA can suggest its function, so we ascertained the subcellular localization of GAS5 through nuclear and cytoplasmic fractionation analysis. GAS5 existed in both the cytoplasm and nucleus, with the predominant fraction in the cytoplasm (Figure 1E,F), which was in accordance with the results of previous studies.^12^

### 3.2 GAS5 inhibited ESCC cell proliferation, migration, and invasion

**Figure 2:**
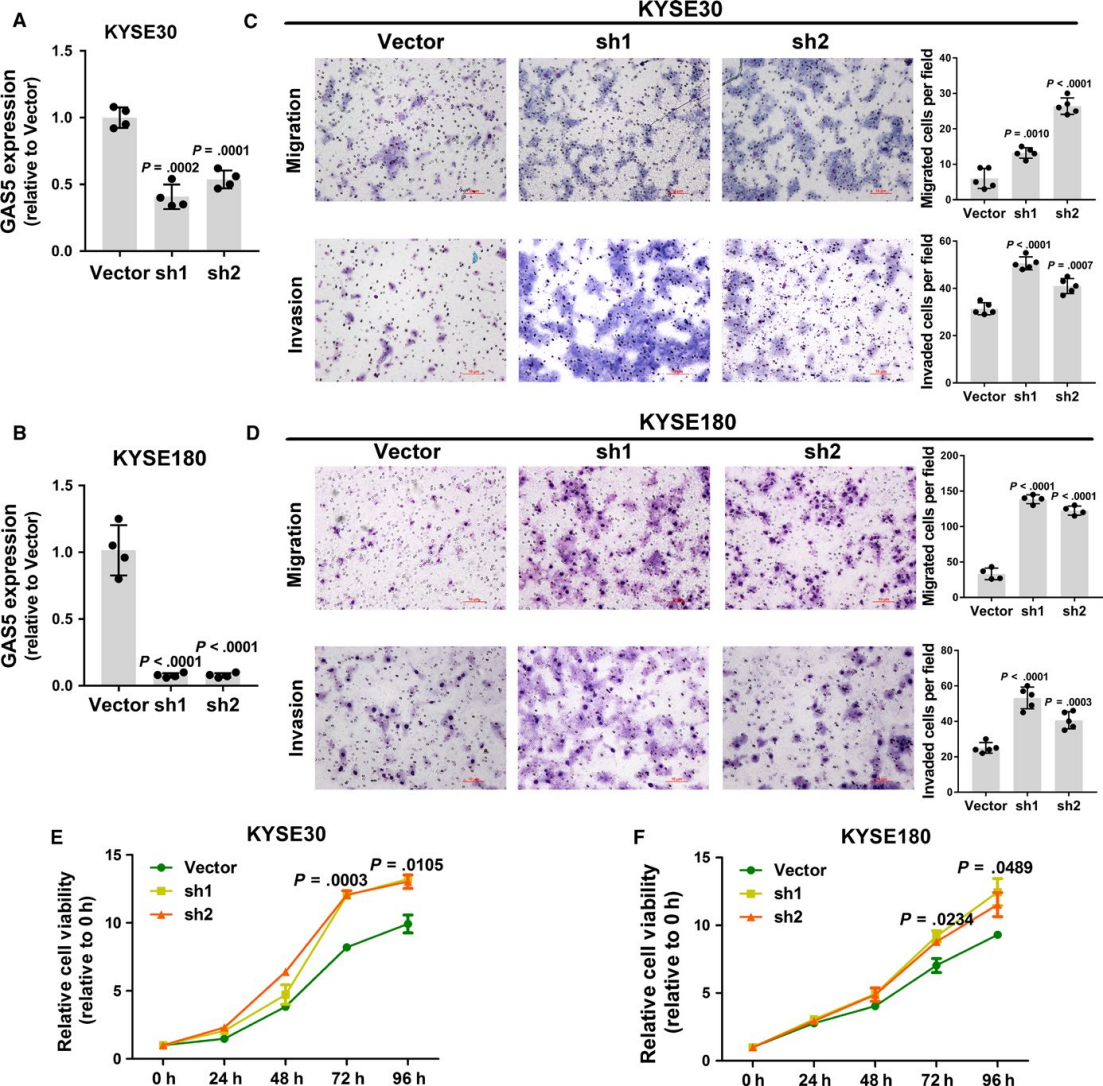
Knockdown of GAS5 promotes ESCC cell proliferation, migration, and invasion. A,B, Validation of GAS5 knockdown efficiency by RT‐qPCR in KYSE30 and KYSE180 cells. *P* values are obtained based on Student's *t*‐test (2 tailed). C,D, Transwell assays of GAS5‐knockdown KYSE30 and KYSE180 cells and vector control cells. The upper panels show the migration results, and the lower panels show the invasion results. Images are shown at 100× magnification. The left histograms are the quantification of transwell assay data. *P* values are obtained based on Student's *t*‐test (2‐tailed). E,F, Proliferation assays of GAS5‐knockdown KYSE30 and KYSE180 cells and vector control cells. *P* values are obtained based on comparing the 2 knockdown groups and control group at the same time point using one‐way ANOVA. The results are the average of at least 2 independent experiments with a minimum of 2 technical replicates per experiment, and the data are presented as the mean ± SD

GAS5, a tumor suppressor lncRNA, has been extensively studied in multiple cancers. GAS5 can inhibit the proliferation and promote the apoptosis of multiple cell types and is a promising diagnostic and prognostic cancer biomarker.^11^, ^12^, ^14^ To study the biological function of GAS5 in esophageal cancer, we silenced GAS5 in 2 esophageal cancer cell lines, KYSE30 and KYSE180 which showed medium GAS5 expression, using lentivirus‐mediated knockdown (Figure 2A,B). First, we compared the cell proliferation of knockdown cells and control cells, and the results showed that knockdown cells exhibited significantly elevated proliferative ability (Figure 2E,F). We next performed transwell assays to evaluate the effects of GAS5 on cell mobility; knockdown of GAS5 increased the migration and invasion of ESCC cells (Figure 2C,D). These results indicated that knockdown of the lncRNA GAS5 could promote ESCC cell proliferation, migration, and invasion.

**Figure 3:**
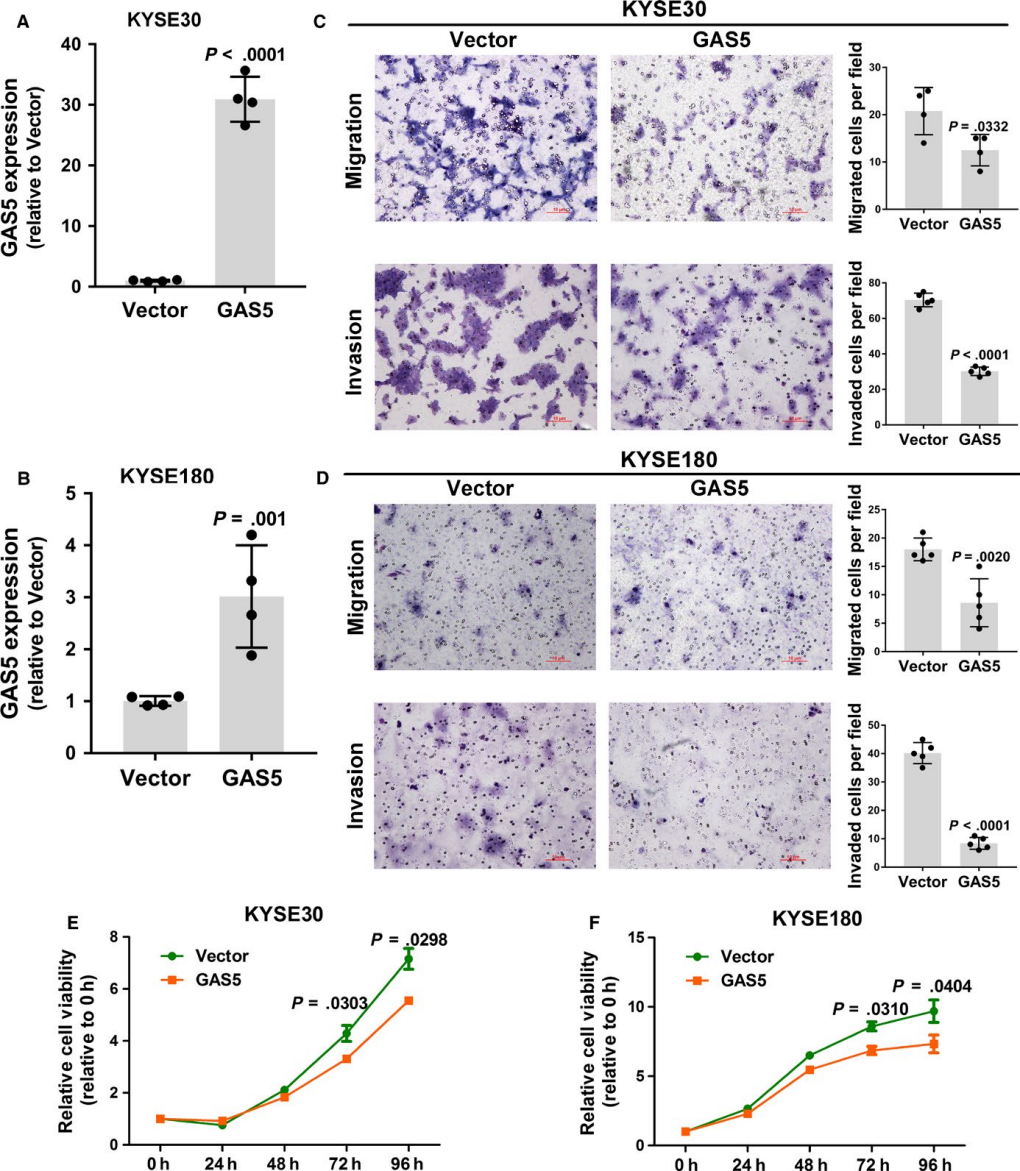
Over‐expression of GAS5 inhibits ESCC cell proliferation, migration, and invasion. A,B, Validation of GAS5 over‐expression efficiency by RT‐qPCR in KYSE30 and KYSE180 cells. C,D, Transwell assays of GAS5‐over‐expressing KYSE30 and KYSE180 cells and vector control cells. The upper panels show the migration results, and the lower panels show the invasion results. Images are shown at 100× magnification. The left histograms represent the quantification of transwell assay data. E,F, Proliferation assays of GAS5‐over‐expressing KYSE30 and KYSE180 cells and vector control cells. The results are the average of at least 2 independent experiments with a minimum of 2 technical replicates per experiment, and the data are presented as the mean ± SD. *P* values are obtained based on Student's *t*‐test (2‐tailed)

To further analyze the effects of GAS5 on ESCC cell viability and mobility, we over‐expressed GAS5 in 2 esophageal cancer cell lines, KYSE30 and KYSE180 (Figure 3A,B). GAS5 over‐expression significantly decreased the proliferation (Figure 3E,F), migration, and invasion (Figure 3C,D) of ESCC cells. Taken together, these results suggested that GAS5 acts as a tumor suppressor via inhibiting ESCC cell proliferation, migration, and invasion.

### 3.3 The lncRNA GAS5 was induced by IFNs via the JAK‐STAT pathway

Saturating cell density and nutrient deprivation can increase GAS5 expression due to post‐transcriptional mechanisms involving the interplay between the mammalian target of rapamycin (mTOR) and nonsense‐mediated decay (NMD) pathways.^15^, ^16^, ^17^, ^18^ Transcriptional mechanisms may control GAS5 expression in differentiating cells,^18^ but the transcriptional regulation of GAS5 expression requires further characterization. To identify potential transcription factors regulating GAS5 expression, we used the online PROMO database to predict transcription factors that may bind to the GAS5 promoter region. The list of transcription factors that potentially bind to the GAS5 promoter included the signal transducer and activation of transcription (STAT) family, which caught our attention (Table S2). IFN mainly functions through the JAK (Janus activated kinase)‐STAT signaling pathway and plays an antitumor role.^19^ IFNs also play an antitumor role in esophageal cancer.^20^, ^21^ We next used another online database, Jaspar, to confirm the interaction between the STAT family and the GAS5 promoter. A list of STAT family members that bind to the GAS5 promoter is provided in Table S3.

**Figure 4:**
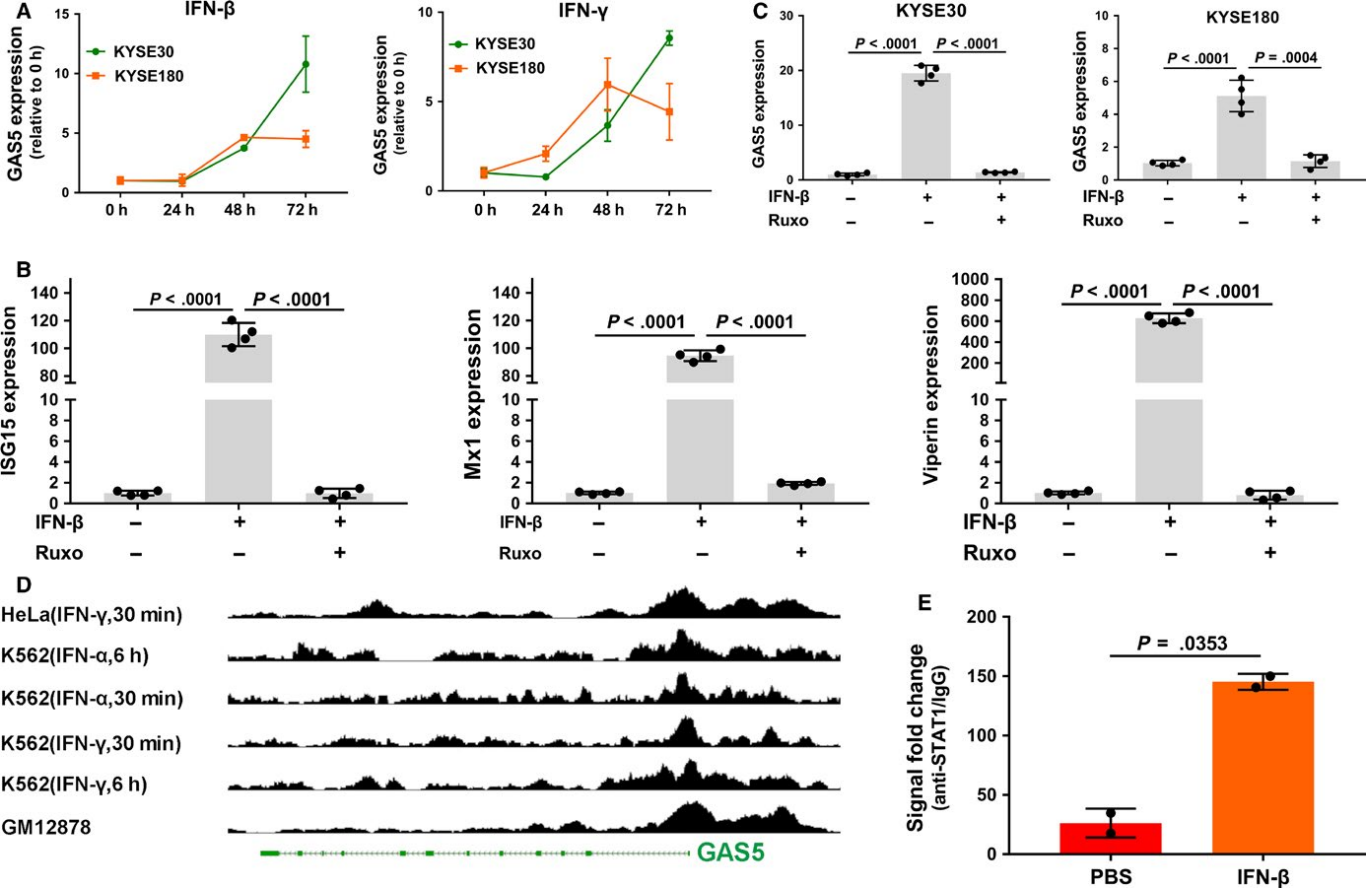
The lncRNA GAS5 is transcriptionally activated by the JAK‐STAT pathway. A, The fold‐change in GAS5 expression in 2 cell lines following IFN‐β and IFN‐γ treatment was detected by RT‐qPCR. The results are shown relative to untreated cells (0 h). B, RT‐qPCR analysis of 3 known ISGs (ISG15, Mx1, and viperin) in the esophageal cancer KYSE180 cell line treated with the JAK inhibitor ruxolitinib (Ruxo). C, RT‐qPCR analysis of GAS5 in esophageal cancer KYSE30 and KYSE180 cell lines treated with the JAK inhibitor Ruxo. The + and − signs at the bottom indicate an added or omitted ingredient, respectively. IFN, IFN‐β; Ruxo, ruxolitinib. D, Visualization of the GSE31477 ChIP‐seq data set from GEO using the UCSC Genome Browser. The results of ChIP with an anti‐STAT1 antibody in different cell types with different IFN stimulation are shown. E, ChIP assay using an anti‐STAT1 antibody in the esophageal cancer KYSE30 cell line. KYSE30 cells were treated with IFN‐β for 6 h prior to the ChIP assay. The results are shown as the mean ± SD. The results are the average of at least 2 independent experiments with a minimum of 2 technical replicates per experiment. *P* values are obtained based on Student's *t*‐test (2 tailed)

To ascertain whether GAS5 is regulated by IFN signaling, we treated ESCC cells with recombinant human IFN‐β (type I IFN) or IFN‐γ (type II IFN). The results indicated that IFN‐β and IFN‐γ both increased GAS5 expression at different time points (Figure 4A). To further confirm that IFN induces the lncRNA GAS5 via the JAK‐STAT signaling pathway, we simultaneously treated esophageal cancer cells with IFN‐β and the JAK inhibitor ruxolitinib. The results indicated that ruxolitinib could abrogate the upregulation of known ISGs, including ISG15 (interferon‐stimulated gene 15), Mx1 (IFN‐induced GTP‐binding protein Mx1) and viperin (Figure 4B) and the lncRNA GAS5 (Figure 4C). Then, we visualized the gene expression omnibus (GEO) ChIP‐seq data set GSE31477, which included antibodies against the STAT family, using the UCSC Genome Browser (http://genome.ucsc.edu/, hg19). The results showed that the STAT1/2/3 transcription factors all have binding peaks around the GAS5 promoter region in several different cell types. The STAT1 results in different cell types are shown in Figure 4D. To further confirm the transcriptional regulation of GAS5 by STATs, we perform ChIP assays using an anti‐STAT1 antibody in esophageal cancer cells. The results indicated that STAT1 could bind to the GAS5 promoter and that IFN‐β stimulation promoted this interaction (Figure 4E). Taken together, these results suggested that the lncRNA GAS5 can be induced by IFNs via the JAK‐STAT pathway.

### 3.4 IFN responses were positively regulated by GAS5

**Figure 5:**
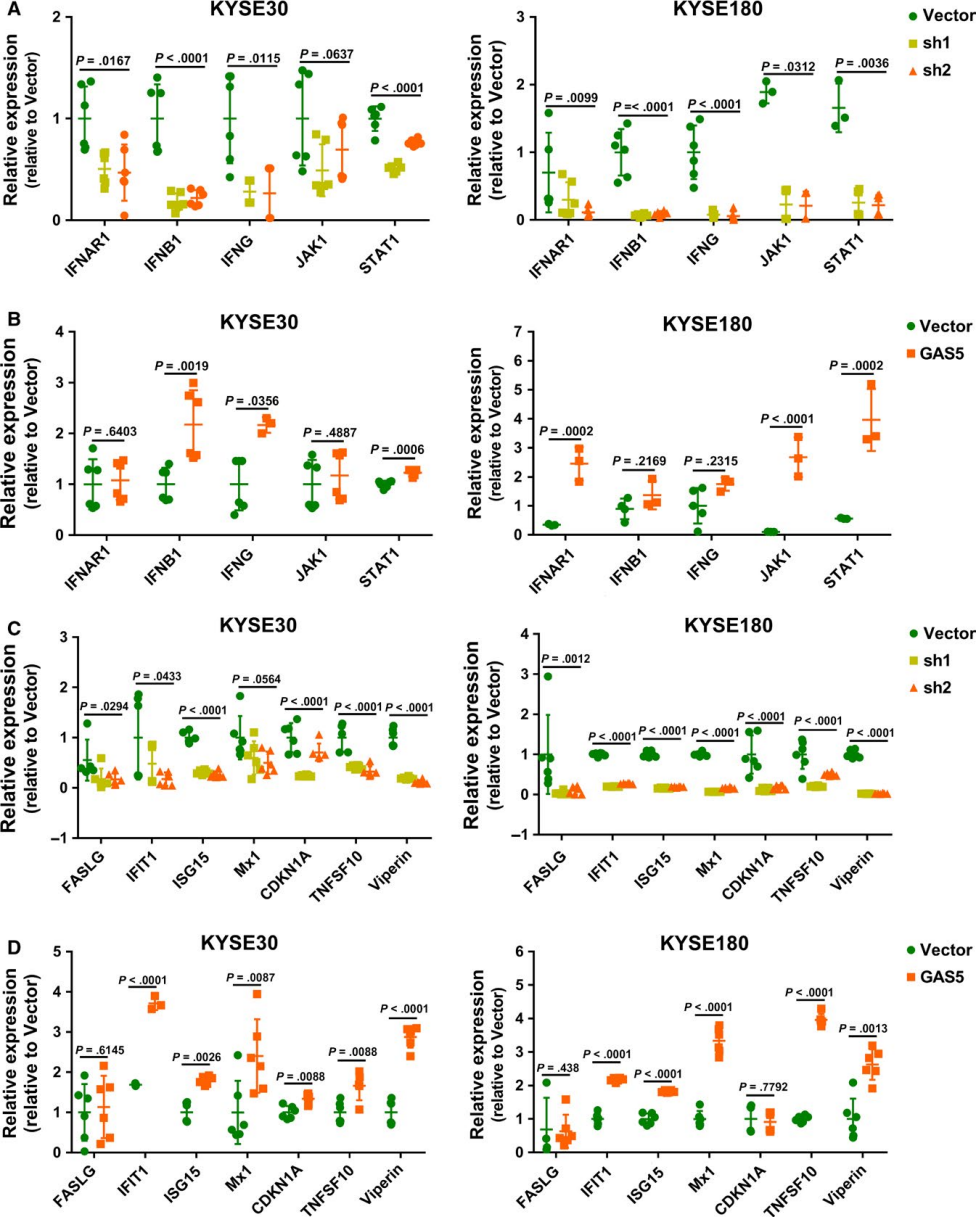
GAS5 is a positive regulator of IFN responses. A, Knockdown of GAS5 in KYSE30 and KYSE180 cells leads to the down‐regulation of genes in the IFN pathway. *P* values are obtained based on one‐way ANOVA. B, Over‐expression of GAS5 leads to the upregulation of genes in the IFN pathway. *P* values are obtained based on Student's *t*‐test (2 tailed). C, Knockdown of GAS5 leads to the downregulation of known ISGs. *P* values are obtained based on one‐way ANOVA. D, Over‐expression of GAS5 leads to the up‐regulation of known ISGs. *P* values are obtained based on Student's *t*‐test (2‐tailed). All results in this figure were obtained by RT‐qPCR, and the data are the average of at least 2 independent experiments with a minimum of 2 technical replicates per experiment. The results are presented as the mean ± SD

**Figure 6:**
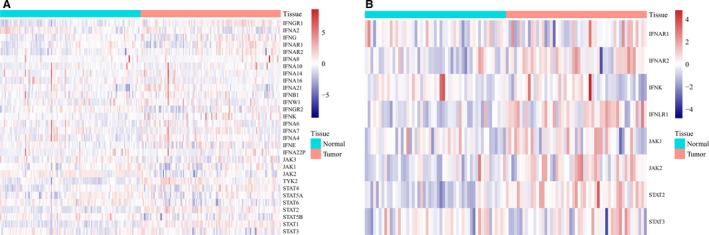
Expression analyses of the interferon signaling pathway. A,B, Heat map of genes in the interferon signaling pathway in the GSE53624 and GSE23400 data sets

Previous studies have indicated that ISGs usually regulate IFN responses via positive or negative feedbacks.^22^, ^23^, ^24^ To evaluate the regulation of IFN responses by GAS5, we detected the mRNA expression levels of IFN pathway‐associated genes, including IFNAR1(interferon‐alpha/beta receptor alpha chain), IFNB1(interferon beta), IFNG (interferon gamma), JAK1 and STAT1, which indicate IFN pathway activity. These genes were downregulated by GAS5 knockdown (Figure 5A) and upregulated by over‐expression of GAS5 (Figure 5B) in 2 esophageal cancer cell lines. To further elucidate the feedback regulation of IFN responses by the lncRNA GAS5, we detected the mRNA expression levels of known IFN‐stimulated genes, including the antitumor effectors FASLG(Fas ligand), CDKN1A(cyclin‐dependent kinase inhibitor 1), and TNFSF10(TNF‐related apoptosis‐inducing ligand)^19^ and the antiviral effectors viperin(Virus inhibitory protein), ISG15(Interferon‐stimulated gene 15), Mx1(Interferon‐induced GTP‐binding protein Mx1) and IFIT1(Interferon‐induced protein with tetratricopeptide repeats 1).^24^, ^25^ These genes were down‐regulated by GAS5 knockdown (Figure 5C) and up‐regulated by over‐expression of GAS5 (Figure 5D). Because GAS5 was over‐expressed in ESCC tissue compared to normal tissue (Figure 1), we analyzed IFN signaling pathway activity in the GSE53624 and GSE23400 data sets. The expression levels of IFN pathway‐associated genes were slightly higher in ESCC tissue than in normal tissue in GSE53624 (Figure 6A) and obviously higher in GSE23400 (Figure 6B); these results were in accordance with the GAS5 expression level in ESCC. Taken together, these results indicated that IFN responses are positively regulated by GAS5 in esophageal cancer.

## 4 DISCUSSION

Esophageal cancer is one of the most common cancers worldwide and is especially common in China; most cases of esophageal cancer are ESCC. The overall prognosis of ESCC is not satisfactory due to the lack of early diagnoses and effective therapies. Up to now, there is no clinically proven therapeutic target in ESCC. A better understanding of the mechanisms of ESCC initiation and progression will enable the identification of novel therapeutic targets for this disease. lncRNAs are ubiquitous in development and disease and are involved in every branch of life.^4^, ^5^ lncRNAs also play a vital role in the initiation and progression of cancer and are promising novel therapeutic targets.^6^, ^26^ Unraveling the functions and mechanisms orchestrated by lncRNAs will provide novel avenues for the management of ESCC.

GAS5, an important tumor suppressor lncRNA, is a promising diagnostic and prognostic cancer biomarker.^14^ Two recent studies indicated that GAS5 is downregulated in human ESCC tissues and inhibits the growth of ESCC cells.^27^, ^28^ In this study, we found that GAS5 was upregulated in ESCC tissues in public data sets and acted as a tumor suppressor gene. In our study, GAS5 expression levels were obtained from a public database, which has a larger sample size than the previously studied ESCC data set. Tumor suppressor genes are usually downregulated in cancer.^29^ However, some tumor suppressor genes are over‐expressed in cancer tissue compared with those in normal tissue; for example, secondary accumulation of mutant p53 has been observed.^30^ Upregulation of GAS5 in ESCC could partly result from regulation by the IFN signaling pathway, which is more active in tumor tissue. The underlying mechanisms in vivo require further study. A recent study showed decreased expression of lncRNA GAS5 predicts poor survival in ESCC.^28^ However, patients with high GAS5 expression and low GAS5 expression showed no significant difference in overall survival in our previous microarray data. Considering decreased GAS5 expression was associated with unfavorable survival in several other cancers,^14^ we infer the non‐significant survival difference between low‐ and high GAS5 expression patients in our study could result from sampling error. Previous studies reported that GAS5 could negatively regulate the migration of ESCC, renal and cervical cancer cell lines,^28^, ^31^, ^32^ whereas there was no reported effect on NSCLC cell migration.^33^ In this study, GAS5 inhibited ESCC cell migration and invasion. These results indicated that GAS5 may have different effects on cell mobility in different cancer types.

GAS5 is post‐transcriptionally regulated by interplay between the mTOR and NMD pathways^12^ and is negatively regulated by miR‐196a in ESCC.^27^ However, the transcriptional regulation of GAS5 was largely unknown. We obtained a list of transcription factors predicted to bind the GAS5 promoter through an online database. This list suggested that GAS5 could be regulated by different signaling pathways. A previous study reported that GAS5 is associated with human embryonic stem cell self‐renewal and is transcriptionally regulated by the pluripotency factors OCT4 and SOX2.^34^ In this study, we demonstrated that GAS5 was induced by IFNs via the JAK‐STAT pathway. IFNs are pleiotropic cytokines that have a long history of involvement in cancer development and treatment. The antitumor roles of IFNs include exerting direct effects on cancer cells and activating immune responses.^19^ Previous studies reported that IFNs and some down‐stream molecules evoke antitumor effects in esophageal cancer via inducing apoptosis or cell cycle arrest.^20^, ^21^, ^35^, ^36^, ^37^ The IFN signaling pathway regulates numerous downstream ISGs, most of which are protein‐coding genes.^23^ Accumulating studies have reported that many lncRNAs are regulated by IFN responses, mainly involving antiviral effects.^38^ This report is the first to show that lncRNAs could mediate the antitumor effects of IFN. Some IFN‐stimulated genes (ISGs), such as FASLG, TNFSF10, and ISG15, were reported to have effects on cancer stem cell formation.^39^, ^40^, ^41^ GAS5 was also reported to control human embryonic stem cell self‐renewal^34^ and suppress malignancy of human glioma stem cells.^42^ Effects of GAS5 on cancer stem cell may need further illustrations, including those on esophageal cancer. ISGs are usually feedback regulators that fine‐tune IFN responses.^23^ In this study, we found that GAS5 was a positive regulator of IFN responses. GAS5 could act as miRNA sponge to positively regulate target genes,^27^, ^34^, ^43^ which may play a role in regulating IFN responses. However, the underlying mechanisms must be more completely elucidated.

Mounting evidence suggests that we could exploit the IFN pathway therapeutically because IFNs both stimulate an antitumor immune response and have a direct effect on cancer cell proliferation and survival.^19^ However, researchers have encountered many obstacles when developing agents to stimulate this pathway, and there is no effective agent currently used clinically. The main issues preventing the clinical use of IFNs are dose‐limiting side effects and unpredictable patient sensitivity. GAS5, a new ISG, adds to the antitumor mechanisms of IFNs and could provide new strategies for predicting IFN sensitivity and developing therapeutic agents.

Crosstalk between the tumor suppressor gene GAS5 and the IFN signaling pathway forms a positive feedback loop that plays an antitumor role in ESCC. The members of this antitumor regulatory loop could represent novel potential therapeutic targets for ESCC.

## CONFLICT OF INTEREST

No potential conflicts of interest were disclosed.
